# Exploring Digital Dermatology: An Analysis of Ichthyosis Content on TikTok

**DOI:** 10.7759/cureus.57401

**Published:** 2024-04-01

**Authors:** Magda S Wojtara, Therese M Guinto, Dorcas K Adebogun, Olivier Uwishema

**Affiliations:** 1 Department of Human Genetics, University of Michigan, Ann Arbor, USA; 2 School of Information Technology, Mapúa Malayan Digital College, Quezon City, PHL; 3 School of Medicine, Virginia Commonwealth University, Richmond, USA; 4 Faculty of Medicine, Karadeniz Technical University, Trabzon, TUR

**Keywords:** skin disease/dermatology, patient-centered, social media education, ichthyosis, tiktok

## Abstract

Background

TikTok is among the most popular social media sites, and its utilization for health information is growing each day. The present study assesses the popularity and quality of the top 100 most-liked videos on TikTok tagged with “#ichthyosis.” This study aims to do so by assessing contributions from physician, and nonphysician sources (such as from patients) to guide healthcare professionals interested in leveraging this platform for public health.

Methodology

A cross-sectional analysis of the top 100 most-liked videos tagged with “#ichythosis” on TikTok as of January 3, 2024, was conducted. The parameters assessed for this study include observable characteristics, content type, and whether the creator was a physician or nonphysician. The quality of the content was measured using the DISCERN scale.

Results

Based on these results, 14 of the top 100 videos were posted by physicians with 1,912,975 as the mean number of views. There were 86 videos posted by nonphysician creators averaging 2,675,341 views. Videos posted by nonphysician creators had a higher average number of views, number of likes, and number of comments but less average saves. Videos made by physicians and educational content had the highest average DISCERN scores, whereas nonphysician, awareness, and personal experience content had the lowest average DISCERN scores.

Conclusions

Physicians are deemed trustworthy, reliable sources of healthcare-related information on TikTok. This study emphasizes the importance of physicians continuing to provide reliable, evidence-based health information on social media platforms such as TikTok.

## Introduction

TikTok is a social media platform with over 1 billion monthly users globally [1]. The platform has a wide range of filters, music options, and lip-syncing templates for its content creators. TikTok users consume and create short videos for about 12-60 seconds [2]. Most creators and consumers on the platform are between 13 and 29 years old [2].

Over the years since its initial launch, TikTok has experienced remarkable growth, and it has now become a significant platform for disseminating timely and helpful information worldwide. Due to its widespread reach and the timeliness of information dissemination, it has been adopted globally as a valuable tool for disseminating health-related information [3]. During the COVID-19 pandemic, the World Health Organization (WHO) utilized TikTok as a means of health communication and education to promote mask use in the public [4]. In China, several well-known health accounts on TikTok are run by recognized elderly doctors of various medical departments and institutions, who use the platform as a tool for patient education [5]. In addition, various public health institutions in China have begun harnessing the power of TikTok and increasingly using it to communicate with citizens on health-related issues [6]. TikTok has also become an essential tool utilized by patients and healthcare providers to enlighten the public about various health conditions, such as ichthyosis and other genodermatoses. 

Genodermatoses are inherited skin disorders that may or may not affect multiple body systems [7]. These skin disorders may be monogenic, polygenic, or chromosomal [8]. An example of a genodermatoses is ichthyosis, a keratinization disorder [9]. This disorder is characterized by cutaneous findings such as hyperkeratosis, xerosis, diffuse scaling, and variable degree of erythroderma resulting from skin inflammation [10]. The onset of symptoms in inherited ichthyosis is usually present at birth or shortly after birth; however, in certain rare cases of acquired forms of ichthyosis, symptoms might be seen in the setting of nutritional deficiency, autoimmune or infectious disease, or malignancy [9]. Underlying the basis of more than 50 different types of ichthyosis are errors in the biosynthesis of epidermal lipids or structural proteins needed for the normal formation and functioning of the skin barrier [11]. Social media platforms such as TikTok are potent tools for spreading information and educating the public about various topics, including health and disease. However, the potential for spreading fake or misleading health information through digital media is a significant threat to public interest [3]. The spread of health misinformation is one of the emerging issues facing public health authorities. The speed at which information can be diffused, ease of accessibility, and difficulty in correcting misinformation make it challenging to control the spread of false information [3]. The ease of use of TikTok makes it possible for any individual to share health-related information without the quality of information or qualifications of the individual sharing the health advice being known or considered [1]. As a result, some health-related information transmitted on TikTok does not meet health standards or regulations [12]. Health-harming industries have in the past paid influential persons online to promote and portray certain health-harming products in a positive light, such as alcohol and vaping. This potential to incentivize individuals to share any form of information, including misinformation on TikTok, is an issue of concern [1]. The spread of health misinformation can lead to delayed proper treatment for patients, thereby adding more casualties to the public health system [3]. More attention must be put into implementing strategies or processes to prevent and report misinformation [1].

The present study aims to assess the popularity and quality of the top 100 most-liked videos tagged with “ichthyosis” on TikTok, comparing content from different sources and assessing the influence of observable characteristics. It is critical to examine the landscape of ichthyosis-related content on TikTok. These findings can inform and guide physicians and other healthcare professionals to make effective use of this platform as a tool for public health education and awareness.

## Materials and methods

This study aimed to assess the popularity and quality of the top 100 most-liked TikTok videos tagged with “ichthyosis” as of January 3, 2024. Thus, a cross-sectional analysis was conducted based on several parameters to ensure relevance and to provide a comprehensive view. For inclusion, the videos had to be hashtagged with “ichthyosis,” and be among the top 100 most-liked videos on the platform as of January 3, 2024. On the other hand, the exclusion criteria excluded videos that were not primarily in English, were shorter than five seconds in duration, or had been removed from the platform for violating TikTok guidelines. There was no selection made based on the country of origin, race/ethnic background, or other factors. 

For each included video, the profession of the creator (physician, nonphysician), content type (i.e., awareness, personal experience, educational content, treatment), and other observable characteristics were assessed. Furthermore, metrics such as the number of video views, likes, comments, and saves were also recorded. This data was manually extracted and organized into a spreadsheet. 

The DISCERN tool was utilized to assess the quality of the health information in these videos. The DISCERN tool is a validated 15-point questionnaire used to evaluate health information concerning treatment options, risks, and benefits [13]. This tool was selected based on previous utility as a valid and reliable qualitative instrument for judging consumer health information [13]. The DISCERN instrument includes a total of 15 main items, including reliability (items 1-8) and treatment choice (items 9-15). The reliability items include explicit aims, aims achieved, relevance to patients, sources of information, currency (date) of information, bias and balance, additional sources of information, and reference to areas of uncertainty [13]. The treatment choice items include how it works, benefits, risk, no options, quality of life, other treatment options, and shared decision-making [13]. A team of two independent reviewers utilized the DISCERN tool and assigned scores from 1 (low) to 5 (high) for each video. Disagreements in scores among the reviewers were resolved by averaging the scores.

## Results

The included videos were comprised of 14 videos by physicians and 86 videos by nonphysicians. Physician-generated TikTok content had a mean DISCERN score of 2.9 with the best quality video scoring a 3.5 and the worst quality video scoring a 1. Most physician content around ichthyosis was posted by dermatologists; however, 21% of these videos were posted by nondermatologist physicians. Interestingly, there was not a large difference in average DISCERN scores between dermatologist and nondermatologist physician content (2.9 and 2.6, respectively). 

Most of the physician-generated videos focused on education and a small percentage focused on the treatment of ichthyosis, while one just focused on awareness. Table 1 shows that the TikTok video content posted by physicians had fewer average views, comments, and likes than nonphysicians but had 1.5 times more saves. 

**Table 1:** Overview of ichthyosis content demographics and quality on TikTok

Content Demographics	Number of Videos (%)	Mean Number of Views	Mean Number of Likes	Mean Number of Comments	Mean Number of Saves	Mean DISCERN Score
Physicians	14	1,912,975	83,924	1,008	6,028	2.9
Nonphysicians	86	2,675,341	146,617	1,614	4,024	1.8
Dermatologist	11	2,425,390	106,582	1,276	7,659	2.9
Nondermatologist	3	34,118	847	26	47	2.6
Type of content: awareness	39	3,975,237	242,253	2,782	4,979	1.8
Type of content: education	13	1,827,011	77,302	945	3,916	2.8
Type of content: personal experience	29	1,070,283	45,524	520	1,461	1.8
Type of content: treatment	16	2,596,477	111,067	920	8,098	2.2

Meanwhile, nonphysician video content had a mean DISCERN score of 1.8 with the best quality video scoring a 4.3 and the worst quality video scoring a 1. The majority of nonphysician video content was regarding personal experience and awareness. Many of the 86 videos were from the same group of content creators, but there was marked diversity in age, skin color, and condition severity among these content creators. 

Of the top 100 videos tagged “#ichthyosis” on TikTok, 95 were less than a minute long and five were greater than a minute with the longest being six minutes. The videos longer than a minute had a mean DISCERN score of 2 compared to a mean DISCERN score of 1.4 for videos shorter than a minute. 

This dataset provides interesting insights regarding the preferences and behaviors of TikTok users regarding ichthyosis content. Physicians’ videos have better DISCERN scores compared to nonphysician content, and there is little difference between scores for dermatologist and nondermatologist physician content quality.

## Discussion

TikTok videos have grown to become a popular medium for conveying health-related information and have also become a source of potential misinformation [1-5]. The videos analyzed in this paper typically fell into the following categories: awareness, education, personal experience, and treatment. Collective scientific understanding of ichthyosis and other genodermatoses has risen over the past few decades [7-11]. As new insights are gained in the realm of disease pathogenesis and genetic origins, treatments are also under development. Many of these treatments aim to improve the quality of life for those living with the conditions. Upon further analysis, some interesting trends arose from the collected data. For instance, despite only a small difference between average DISCERN scores for dermatologists and nondermatologists physician content (2.9 and 2.6, respectively), dermatologist content received much more user interaction. The average number of views for dermatologist content tagged #ichthyosis was 2,425,390 compared to 34,118 for nondermatologist physicians. Likewise, the average number of likes for dermatologist-generated content was 106,582, while nondermatologist physician content averaged 847. Furthermore, the dermatologist TikTok videos averaged 1,276 comments compared to 26 for nondermatologist physician videos. Perhaps most striking, however, was the difference in the number of video saves. Dermatologist content was saved by an average of 7,659 users as opposed to an average of 47 users saving nondermatologist physician content. 

Of the relevant posted content, videos in the awareness and personal experience categories had the lowest average DISCERN scores (both of 1.8). It is important to note that patient-generated content may not have the intention to share specific medical details of their condition and tends to be more broad and less technical. This can be contrasted with the video category with the highest average DISCERN score: education (2.8). The primary intent behind the educational TikTok videos was to inform, and thus, these videos tended to align more with the DISCERN questionnaire criteria. However, the videos in the awareness category had the highest mean number of views, likes, and comments. This type of content also often contained videos of patients with severe ichthyosis symptoms or content by physicians. The video category with the highest number of saves was treatments for ichthyosis. This was likely, in part, due to patients and family members wanting to explore avenues to improve symptoms. 

Concerning other characteristics of the videos, qualitative descriptors were also collected during the initial data collection stage. From these descriptors, several other insights emerged. There is representation in both patient and nonpatient TikTok videos of several types of ichthyosis. These include harlequin ichthyosis, lamellar ichthyosis, epidermolytic ichthyosis, and ichthyosis vulgaris. There were 29 different creators with videos in the top 100 tagged with #ichthyosis, either patients with ichthyosis themselves or sharing the experiences of a close family member. Many of these creators, however, did not have their accounts focusing on their condition and instead were often replying to comments on other videos to explain their condition and experiences. Among creators who predominantly posted ichthyosis content, the one with the most engagement had 6.8 million likes on their videos and more than 250,000 followers on the platform. 

Limitations

TikTok rose rapidly in its utilization in part because its interface is relatively simple and easy to navigate. The interface consists of several “feeds” where videos are suggested based on the accounts followed and content engaged with. TikTok and other social media platforms have, thus far, not been thoroughly explored regarding the promotion of microlearning (defined as the acquisition of knowledge in small units) [14]. Microlearning through simply watching these videos on the platform can enhance user understanding and learning of the topic. Even among learners, in a prior study, participants were shown to evaluate videos on complementary content as positively (no statistically significant difference) as videos with associated exam-related content [14]. TikTok is predominantly a platform that differentiates itself by disseminating short, brief video content. Recently, TikTok extended the maximum video duration, which allowed creators to better express and explain their content [15]. 

However, these are also inherently drawbacks to the platform as well as its largest assets. The hypercurated content appearing on many individuals’ accounts means that they may be missing out on viewing new, informative content that may become interesting in the future. Furthermore, many users may take content on social media at face value, especially if posted by a source perceived to be trustworthy (doctor or patient with the condition). Microlearning is largely passive, and full education on a topic should be expected only if an individual follows up on this content with further exploration of high-quality materials. Many laypeople, however, are not aware of where to find reliable, high-quality further reading and viewing [15]. Even if they do find articles after searching, they are often not as accessible to understand as short-form TikTok video content [15]. Furthermore, short videos are well suited for providing a few sentences worth of information, but health materials especially can benefit from longer-form explanations. From the standpoint of content creators, striking a balance between these two extremes can likely be challenging. 

It is also important to consider that different but similar searches may have yielded different results. For example, utilizing a more specific search term such as “#ichthyosisvulgaris” is more likely to yield even more tailored, specific content. Our search was also limited to English-language videos. Likewise, using a broader search term such as “#geneticdisease” is likely to yield more broad, overarching content. The search results were up-to-date as of January 3, 2024; however, more videos are posted on the platform daily, and these newly posted videos may have different DISCERN scores or vary in other ways. 

While the DISCERN tool is widely considered to be an appropriate tool for assessing video quality, there are additional measures that were not utilized in this present study but that could increase the reliability and validity of any findings. For instance, the Medical Information and Content Index (MICI) is specifically used to analyze the content of informative videos [16]. Another option is utilizing the Global Quality Score (GQS) metric which also assesses the quality and usefulness of video content [16]. In general, there are some limitations inherent to the DISCERN tool. This includes that the inherent subjectivity is required for rating certain criteria [13]. However, the findings demonstrate that the instrument can be applied by experienced users and providers of health information to discriminate between publications of high and low quality [13]. Since there is subjectivity in this analysis, this is more of a qualitative evaluative tool. Future studies can explore additional statistical analyses, apply this methodology to other platforms, and perform additional analyses to gain a more comprehensive understanding of the quality of healthcare content on social media platforms. 

The TikTok algorithm is not fully public; however, there are some features of videos that make content more likely to be recommended and viewed on the platform. Videos with high-quality sound and video and that use relevant keywords are more likely to be recommended and subsequently engaged with by TikTok users. Furthermore, content that adheres to current trends such as by using a trending audio/song is also more likely to be recommended and have increased video engagement. Therefore, there may be other videos posted by physicians and nonphysicians that provide high-quality educational content but may be outside the top 100 posts tagged #ichthyosis due to this.

## Conclusions

TikTok's ease of use, speed in information dissemination, and popularity make it a good platform for health information dissemination. Healthcare professionals such as physicians can harness its potential to provide concise, easily understood information to the public seeking health education. Patients may also benefit from seeing these videos and thereby finding others living with similar conditions. 

Based on the analysis done in this study on the quality of the top videos on TikTok on the subject of ichthyosis, it was shown that physician-generated videos were of higher quality, as seen by the higher DISCERN scores as compared to nonphysician-generated videos. Emphasis or priority needs to be placed on fact-checking and assessing the quality, veracity, and credibility of videos posted for health education to prevent misinformation; however, videos made by physicians in different specialties will continue to be a good reference for the majority of the platform's users seeking health information. Future studies should explore the quality of healthcare content across platforms and for other conditions. 
